# AI-Based Dose Compliance of Secondary Organs at Risk in Head and Neck Cancer Radiotherapy

**DOI:** 10.3390/diagnostics16111748

**Published:** 2026-06-05

**Authors:** Ioana-Claudia Costin, David C. Marcu, Loredana G. Marcu

**Affiliations:** 1 Bihor County Emergency Clinical Hospital, 410167 Oradea, Romania; ioanaclaudiacostin@yahoo.com; 2 Faculty of Informatics & Science, University of Oradea, 410087 Oradea, Romania; 3 Faculty of Electrical Engineering & Information Technology, University of Oradea, 410087 Oradea, Romania; david.marcu@uoradea.ro; 4 Allied Health and Human Performance, Adelaide University, Adelaide, SA 5001, Australia

**Keywords:** treatment planning, deep learning, autosegmentation, artificial intelligence, performance metrics

## Abstract

**Objective**: Alongside evaluating the geometric and dosimetric performance of an AI auto-segmentation algorithm on conventional (primary) organs at risk (OARs), the aim of this work was to highlight its added advantages in terms of the dosimetric evaluation of secondary OARs, which are commonly under-reported despite their influence on overall toxicity. **Methods**: The study included 50 head and neck cancer patients, with volumetric modulated arc radiotherapy (VMAT) plans created based on both manual contouring and auto-contouring using a commercially available auto-segmentation tool. Quantitative assessment of auto-contouring was undertaken on 10 primary OARs using geometric performance metrics (the Dice Similarity Coefficient (DSC), Hausdorff distance (HD), sensitivity, and precision) as well as dosimetric differences between manual vs. auto-segmentation. Once the algorithm was validated on primary OARs, a dosimetric assessment of 29 secondary OARs delineated by the tool was conducted. **Results**: Manual contouring required 21.20 ± 2.25 min, while 9.25 ± 1.42 min were needed for auto-segmentation with adjustment. Dosimetric differences were found for the brainstem and the right submandibular gland. The mandible presented the maximum HD value of 24.09 ± 18.83 mm. Sensitivity, precision and DSC ranged from 0.77 to 0.93. For the 29 secondary OARs, 1500 dosimetric values were collected. Of these, 8.5% exceeded the dose constraints. The most impacted OAR was the constrictor muscle, exceeding the constraint in 56% of cases, while the glottis came in second. The dosimetric results also raised concerns regarding organs without defined dose constraints. **Conclusions**: When validated for primary OARs, auto-segmentation offers a more comprehensive dosimetric evaluation of secondary OARs to further reduce the dose to sensitive structures.

## 1. Introduction

Segmentation of organs at risk (OARs) is a key step in radiotherapy planning, as the accurate delineation of such organs ensures that prescribed radiation doses maximize tumor control while minimizing damage to adjacent critical structures, directly influencing treatment outcomes and toxicities. Manual OAR contouring is labor-intensive and subject to significant inter- and intra-observer variability caused by differences in anatomical interpretation, contouring experience and image quality, which can lead to dosimetric inconsistencies and prolong treatment planning workflows [1,2]. Automated segmentation techniques have therefore emerged as key technologies aiming to standardize contouring, reduce clinician workload, and support adaptive and personalized RT planning [3,4,5].

Over the last decade, deep learning (DL) has revolutionized auto-segmentation, outperforming traditional atlas-based approaches in both accuracy and efficiency [6,7]. In multi-site cohorts spanning the head and neck, thorax, and pelvis, convolutional neural network-based segmentation yielded statistically superior geometric accuracy (the Dice similarity coefficient—DSC, Hausdorff distance—HD) across most OARs, as well as a reduction in editing time in clinical workflows [8]. In studies involving the head and neck, central nervous system and prostate OARs, DL models demonstrated performance that approached or matched the variability among experienced clinicians, while dramatically reducing contouring time [9]. Clinical deployment studies show that auto-segmentation algorithms can be routinely integrated into planning workflows, with surveys of radiation therapists and oncologists indicating overall satisfaction and a decreased need for extensive contour editing [10].

In head and neck cancer radiotherapy, the delineation of organs at risk remains a major challenge due to the complex anatomy, poor soft-tissue contrast, and high inter-observer variability [11,12]. Manual delineation is time-consuming and subject to significant inconsistencies, particularly for small or ill-defined structures such as the brachial plexus, cochlea, optic apparatus, and pharyngeal constrictor muscles, which can lead to clinically relevant differences in dose distribution and toxicity prediction [11,12,13,14].

The goal of the current work was to evaluate the integration of auto-segmentation within the clinical workflow for the dosimetric evaluation of secondary (under-reported) organs at risk, i.e., organs that are not commonly delineated and evaluated in the context of head and neck cancer. The performance of the algorithm in terms of time efficiency, geometric performance and dosimetric accuracy was assessed to determine its suitability for the above-stated goal. Unlike most previous studies focusing primarily on the geometric validation of deep learning-based segmentation for standard OARs, the present work extends the analysis to a larger set of secondary organs, providing a comprehensive dosimetric evaluation that is rarely addressed in the existing literature. A dosimetric evaluation was found to be essential as geometric similarity alone does not fully capture the clinical relevance of segmentation differences; even small contouring variations may lead to significant changes in dose distribution to critical structures.

## 2. Materials and Methods

### 2.1. Patient Selection

This study enrolled and retrospectively evaluated 50 patients (with a mean age of 64.24 years) with confirmed head and neck cancer who were treated between 2024 and 2025, using VMAT (volumetric modulated arc radiotherapy) with a 6 MV Elekta Synergy Platform Linear Accelerator equipped with an Agility multileaf collimator (Elekta, Stockholm, Sweden). The main patient characteristics are presented in Table S1.

Patients were selected randomly, without considering tumor stage, gender or age. However, patients were excluded if they: (1) were treated for thyroid cancer (due to distinct disease-specific anatomical distribution), (2) had received a different fractionation scheme (i.e., with a fraction dose greater than 2 Gy and a total dose other than 70 Gy; to ensure treatment uniformity), and (3) had been resimulated and replanned, as the summation of plans could lead to discrepancies in OAR dosimetry and compromise consistency between treatment plans.

### 2.2. Manual Segmentation

The computer tomography (CT) images obtained during patient positioning simulation were imported into the treatment planning system (TPS) Monaco 6.2.2.0 (Elekta, Stockholm, Sweden) for manual segmentation. According to the internal protocol, the following OARs were contoured, regardless of the tumor localization: the brainstem, spinal cord, esophagus, thyroid gland, submandibular glands, parotid glands, oral cavity and mandible.

The 10 OARs were delineated by both a senior radiation oncologist and an experienced medical physicist. The reference segmentation (ground truth) was defined as the overlap of the independently delineated contours. The volume was generated using the “intersect” function available in the TPS. No formal assessment of inter-observer variability was performed; therefore, the reference standard represents a consensus-derived approximation.

Additionally, the time required for contouring all 10 OARs was recorded independently by both the physicist and the physician, with the mean value being reported in the current study.

### 2.3. Auto-Segmentation

The CT images delineated in the TPS were imported into the Mediq RT platform (Synaptiq Collective Intelligence, Cluj-Napoca, Romania [15]) for the auto-segmentation of all OARs located near the tumor volume. The architecture of the algorithm is based on the U-Net framework, with 50 million trainable parameters. The model was trained on approximately 7000 CT scan images, demonstrating its capacity to perform complex segmentation and detection tasks in medical imaging.

The AI algorithm delineated 39 OARs from the CT images of the whole cohort. During auto-segmentation, the amount of time required to contour the 39 OARs was recorded. The following organs at risk were automatically segmented: the brain, temporal lobes, brainstem, optic chiasm, pituitary gland, internal ears, cochleas, eyes, lenses, optic nerves, lacrimal glands, cervical vertebrae, sublingual glands, submandibular glands, parotid glands, thyroid gland, larynx, larynx-supraglottis, glottis, trachea, esophagus, clavicles, temporomandibular joints, mandible, oral cavity, constrictor muscle, brachial plexus and spinal cord.

To remain consistent with the study’s objectives, the integration of the AI algorithm into routine clinical practice was evaluated by having the physicist compare, manually review and adjust the segmentations generated by the algorithm against the manual segmentations (i.e., adjusted auto-segmentation) for the ten OARs, while recording the time required for adjustments. Each organ was carefully evaluated by assigning adjustment levels (low, medium, and high) based on the extent of manual correction required, with corresponding scores (1 = low, 2 = medium, 3 = high). These levels were determined through expert consensus to reflect minimal, moderate, and extensive adjustments.

An adjustment coefficient was then calculated to quantify the overall degree of correction required for each organ across the patient cohort, using the following expression:

$$\mathrm{Adjustment}\;\mathrm{coefficient}\;=\;\frac{\mathrm{Final}\;\mathrm{adjustment}\;\mathrm{score}}{\mathrm{Reference}\;\mathrm{score}}$$

where the final adjustment score represents the sum of individual adjustment scores for a specific organ across all patients. The reference score was considered to be 50, corresponding to the number of patients included, representing a scenario in which only minimal adjustments (score 1) would be required per patient. Clinically, a lower adjustment coefficient indicates that the auto-segmentation closely matches the manual ground truth (small corrections performed on only some of the CT slices within the volume), meaning reduced manual contouring time, decreased editing workload for clinicians, and improved workflow efficiency. The ideal situation would require no adjustment at all, meaning that the segmentation produced by the AI algorithm would perfectly correspond with the manual segmentation for each patient. This situation is impossible to achieve in practice, especially considering that the ground truth itself is defined within intra- and inter-observer variability.

### 2.4. Treatment Planning and OARs Dosimetry

Two-dose prescriptions were used for the treatment plans: (1) an integrated boost (56 Gy, 63 Gy and 70 Gy in 35 fractions) and (2) a sequential boost (50 Gy in 25 fractions, 60 Gy in 30 fractions and 70 Gy in 35 fractions) (see Table S1). All treatment plans were performed using the VMAT technique, employing 1–2 arcs with 6 MV energy, a minimum segment width of 1 cm and high fluence.

The OARs were divided into two categories: the conventional (primary) OARs included the organs that are routinely delineated and were contoured by the physician, physicist and AI algorithm (brainstem, spinal cord, esophagus, thyroid gland, submandibular glands, parotid glands, oral cavity and mandible), while the under-reported (secondary) OARs comprised the organs that are not routinely contoured and were delineated only by the AI algorithm (brain, temporal lobes, optic chiasm, pituitary gland, internal ears, cochleas, eyes, lenses, optic nerves, lacrimal glands, cervical vertebrae, sublingual glands, larynx, larynx-supraglottis, glottis, trachea, clavicles, temporomandibular joints, constrictor muscle, brachial plexus). Dose constraints are presented in Table 1 and are in agreement with the DAHANCA 2025 radiotherapy guideline version 2 and Bisello et al. 2022 [16,17].

For the OARs without a defined dose constraint, the mean dose (for sublingual glands) and maximum dose (for sublingual glands, cervical vertebrae and clavicles) were recorded. Additionally, since the prescribed dose was 70 Gy and some of the secondary OARs were located adjacent to the tumor volume, 90% of the prescribed dose was also evaluated for these OARs, as this was considered relevant as a magnitude for possible correlations with side effects.

### 2.5. Toxicity Assessment

Acute toxicities, specifically xerostomia (dry mouth), dysphagia (swallowing difficulty), and odynophagia (painful or burning sensation during swallowing), were evaluated at the end of radiotherapy using the Common Terminology Criteria for Adverse Events (CTCAE) version 6 [18]. Only Grade 1 (mild symptoms) and 2 (moderate symptoms requiring dietary modification/intervention) toxicities were observed and recorded. Toxicity data were subsequently correlated with the radiation dose delivered to the corresponding OARs (xerostomia → sublingual glands; dysphagia → constrictor muscle, larynx-supraglottis and glottis; odynophagia → constrictor muscle, larynx-supraglottis and glottis) in order to assess the clinical significance of inter-patient dosimetric variations and their potential impact on observed toxicities. This is particularly relevant given that such structures were not routinely contoured in our standard clinical practice, highlighting the role of auto-segmentation in enabling a more comprehensive dosimetric evaluation. Correlation coefficients (r) resulting from the Pearson correlation statistical test were interpreted as weak for values < 0.4, moderate for values between 0.4 and 0.7, and strong for values > 0.7.

### 2.6. Performance Metrics

To assess the auto-segmentation efficiency and performance of the primary OARs, geometric metrics (DSC, HD, sensitivity, precision) were calculated using the manually delineated volume (M) and the AI-generated volume (A). The overlap between the manually delineated and the automatically delineated volumes (M ∩ A) was calculated within the TPS.

The DSC quantifies the overlap between two objects (0—no overlap, 1—perfect overlap) using the formula [19]:

$$\mathrm{DSC}\;=\;\frac{2\;\times \;\mathrm{volume}\;(M\;\cap \;A)}{\mathrm{volume}\;M\;+\;\mathrm{volume}\;A}$$

The HD is the metric that quantifies the distance between the surface points of two objects [20]. The maximum distance calculates the largest distance between corresponding points on the contours of the two objects, accounting for contouring errors. The 95% distance (95% HD) measures the distance between the surface points of object M and their corresponding points in object A, ignoring the top 5% largest errors, which provides a clinically relevant measure of contour accuracy less affected by outliers [21]. The HD was calculated using 3D Slicer 5.8.1 with the Segment Comparison extension. Lower values correspond to a higher degree of agreement between the two objects [22].

Sensitivity represents the rate at which objects were correctly classified, focusing on how much of the manual segmentation is correctly identified by the automatic segmentation [23]. Values vary between [0, 1], where a value of 0 indicates no identification between the segmentations, while a value of 1 indicates perfect identification.

$$\mathrm{Sensitivity}=\frac{\mathrm{volume}\;(M\;\cap \;A)}{\mathrm{volume}\;A}$$

Precision measures the volume of the automatic segmentation that correctly matches the manual segmentation [23]. A value of 0 indicates no overlap, while a value of 1 indicates a perfect match.

$$\mathrm{Precision}=\frac{\mathrm{volume}\;(M\;\cap \;A)}{\mathrm{volume}\;M}$$

If the auto-segmentation misses parts of the manual segmentation, sensitivity will be low, even if precision remains high, highlighting their independent relationship.

## 3. Results

### 3.1. Time Quantification

Since one of the objectives of this study was to evaluate the efficiency of auto-segmentation, Table 2 presents the mean values and standard deviations of the segmentation and adjustment times.

It was observed that the auto-segmentation (0.33 ± 0.11 min) reduces the delineation time by a factor of 38 compared to the manual process (21.20 ± 2.25 min). However, auto-segmentation adjustment and verification were employed to simulate its practical use, which leads to an increase in time to approximately 10 min. Even so, as shown in Table 3, the total time spent is still 2.27 times less when compared to manual delineation.

### 3.2. Segmentation Adjustments

**Figure 1:**
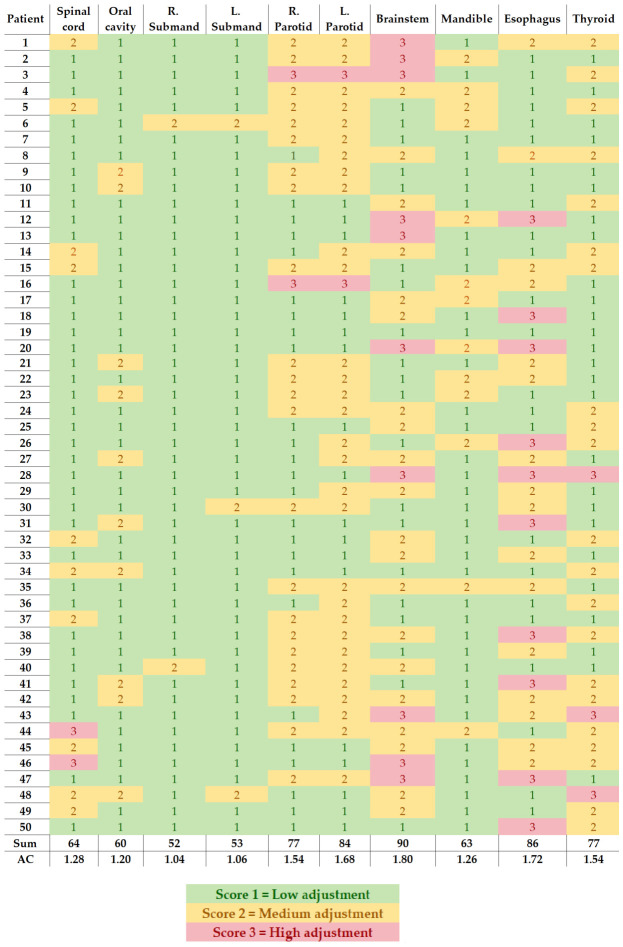
Heatmap of the auto-segmentation adjustment and organs at risk score (Abbreviations: R. Submand = right submandibular gland; L. Submand = left submandibular gland; R. Parotid = right parotid; L. Parotid = left parotid; AC = adjustment coefficient).

As mentioned above, the auto-segmentation of the primary OARs was verified and adjusted according to the manual segmentation, setting adjustment levels and scores for a more precise delineation and identification of OARs. Figure 1 presents the primary OARs scores and a heatmap of the adjustment levels.

As seen in Figure 1, the submandibular glands presented values strikingly close to the reference threshold (52 for the right submandibular gland and 53 for the left submandibular gland). In contrast, the brainstem and esophagus showed significantly higher values, 90 and 86, respectively, indicating poorer performance in these particular organs.

Consequently, the highest adjustment coefficients were also identified in the brainstem (1.80) and esophagus (1.72), meaning that the brainstem and esophagus had to be adjusted 80% and 72% more compared to organs with an ideal reference score. Meanwhile, the submandibular glands showed minimal adjustments of 4% (right submandibular gland: 1.04) and 6% (left submandibular gland: 1.06).

**Figure 2:**
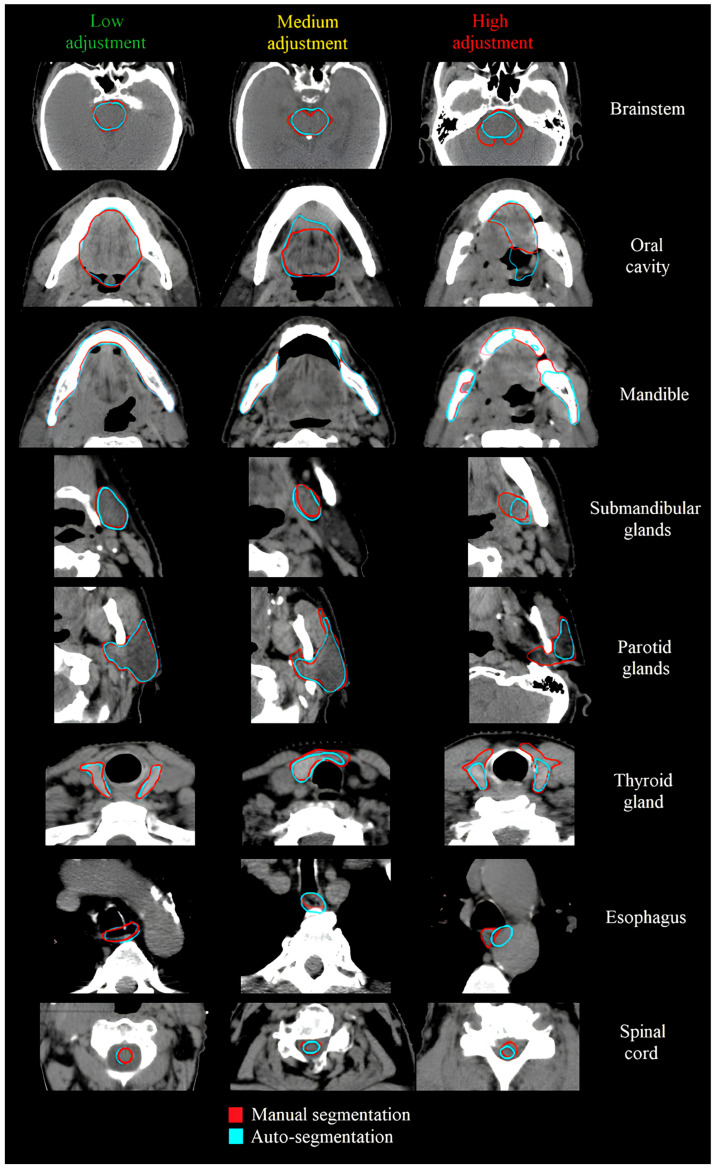
Adjustment levels of the auto-segmentation compared to manual segmentation of primary organs at risk.

Figure 2 shows examples of low, medium and high discrepancies between the manual and auto-segmentation for each of the primary OARs. The most notable differences between the two segmentation approaches are observed in the high adjustment column, where the algorithm demonstrates inhomogeneities (mandible), delineates unintended organs (esophagus) or fails to adequately segment certain organs (parotid glands, submandibular glands, brainstem and thyroid gland).

### 3.3. AI Algorithm Performance and Metrics Quantification

The performance of the auto-segmentation can also be evaluated through performance metrics. The mean and standard deviation of the maximum HD, 95% HD (Figure S1 from Supplementary data), DSC, sensitivity and precision (Figure S2 from Supplementary data) for the ten primary OARs are presented in Table 3.

The poorest performance of the algorithm was observed for the mandible, which was the organ for which the 95% HD had the highest value (6.32 ± 8.40 mm). The best performance was observed for the submandibular glands (right 3.17 ± 1.50 mm and left 3.87 ± 2.91 mm).

### 3.4. Dose Differences Between Manual and Auto-Segmentation

**Figure 3:**
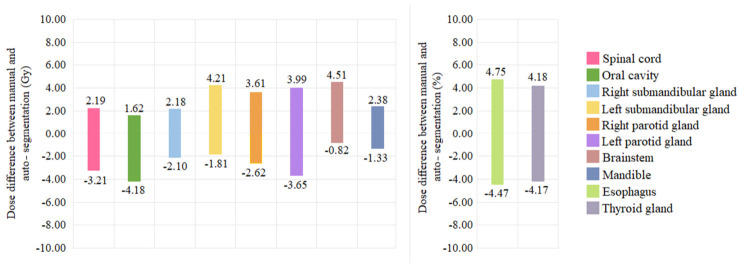
Range of dose difference between manual and auto-segmentation for the primary organs at risk.

The difference in doses between the manually and auto-segmented organs was calculated by subtracting the doses received by the auto-delineated volume from the manually delineated volume, with the manual contours serving as the reference or ground truth. Figure 3 presents the dose difference range between the manual and auto-segmentation.

The highest positive dose difference between the manual and auto-segmentation was observed in the brainstem (4.51 Gy), while the highest negative difference was observed in the oral cavity (−4.18 Gy).

For organs with dose constraints expressed as a percentage volume to receive a certain dose, the highest negative dose difference between the two segmentation methods was observed in the esophagus (−4.47%), indicating that the dose from the manual segmentation was lower than that from the auto-segmentation. The highest positive dose difference was also observed in the esophagus (4.75%) meaning that the dose from the manual delineation was higher than that from auto-delineation.

### 3.5. Correlations Between Metrics and Dose Differences

Correlations were calculated between the dose differences in the primary OARs, segmented manually and automatically, and all the performance metrics. Table 4 presents the Pearson correlation values.

A strong negative correlation was observed for the oral cavity for both the maximum HD (r = −0.747) and the 95% HD (r = −0.795), indicating that a larger distance between the contour points generated by the two segmentations results in a smaller dose difference, with the doses from the manual delineation being lower than those from the auto-segmentation.

Furthermore, the right parotid gland showed a moderate positive correlation with both the DSC (r = 0.511) and sensitivity (r = 0.613), while the left parotid gland showed a moderate positive correlation only with sensitivity (r = 0.476). Additionally, the brainstem presented a moderate negative correlation with precision (r = −0.611), meaning that a higher value of this metric could reduce the dose difference between the two segmentations.

### 3.6. Secondary OARs

Organs at risk that are not commonly evaluated during the radiotherapy planning process for head and neck cancer (according to the internal protocol) are categorized as secondary or under-reported OARs. These, however, were delineated by auto-segmentation, thereby allowing for dosimetric assessment.

Table 5 presents the mean and highest doses received by the secondary OARs, while Table S2 presents an overview of the doses for individual patients. Among the secondary organs at risk, some were included in the tumor volume during planning and delivery, either partially or fully. Of the 50 patients evaluated in this study, 25 had the constrictor muscle partially included within the tumor volume. Consequently, while the reported mean dose remained just under the dose constraint (Dmean < 50 Gy; reported 49.90 ± 6.59 Gy), the highest observed dose exceeded this value (61.71 Gy).

It was observed that the dose constraint was exceeded for the larynx and glottis (larynx V50 < 25%: 27.15 ± 39.03%; glottis Dmean < 45 Gy: 51.53 ± 15.02 Gy), despite the fact that the dose calculations for the mean dose excluded patients with laryngeal and glottic tumors. Additionally, it was noted that although the mean values for the internal ears, optic nerves, larynx-supraglottis and brachial plexus did not exceed the dose constraints, the maximum recorded values surpassed them (internal ears Dmax < 50 Gy: left 59.04 Gy, right 69.79 Gy; optic nerves Dmax < 54 Gy: left 56.79 Gy, right 58.08 Gy; larynx-supraglottis Dmax < 66 Gy: 77.15 Gy; brachial plexus Dmax < 66–70 Gy: 70.43 Gy).

While some organs at risk, such as the sublingual glands, clavicles and cervical vertebrae, do not have specific dose constraints (using ALARA—as low as reasonably achievable), they still received significant mean doses when a prescribed dose of 70 Gy was used (see Table 5 and Table S2).

### 3.7. Assessment of Treatment-Related Toxicities for Secondary OARs

Weak-to-moderate correlations were observed between the radiation dose to the secondary organs at risk and treatment-related toxicities (Table S3 from the Supplementary data). Xerostomia showed a moderate correlation with sublingual gland Dmean (r = 0.449) and a weak correlation with the Dmax (r = 0.347). Regarding swallowing-related toxicities, weak correlations were identified between dysphagia and the dose to the constrictor muscles (r = 0.252) and the supraglottic larynx (r = 0.241). Odynophagia demonstrated slightly higher correlations with the supraglottic larynx dose (r = 0.388) and the constrictor muscle dose (r = 0.280). No strong correlations were observed between the dosimetric parameters and the evaluated toxicities.

## 4. Discussion

Manual contouring is increasingly being replaced by deep learning-based segmentation algorithms to improve efficiency, consistency and accuracy in delineating volumes of interest. In addition to the aforementioned advantages, auto-segmentation algorithms enable the delineation of a much larger number of adjacent organs than is normally evaluated within local protocols, which are often under-represented in the current literature despite their potential clinical relevance. The current paper focused on this latter aspect to highlight the clinical value of auto-segmentation in terms of patient outcomes and quality of life by extending the analysis to the secondary organs at risk and their dosimetric impact.

### 4.1. Contouring Time

The auto-segmentation process demonstrated greater efficiency than manual segmentation in terms of the time required for the delineation of the ten primary OARs. In our study, the mean manual contouring time was 21.20 ± 2.25 min, compared to 0.33 ± 0.11 min for auto-segmentation. After the verification and adjustment of the AI contours, the total time was 9.25 ± 1.42 min. Similarly, Chen et al. (2021) reported an adjusted AI time of 13.10 ± 3.14 min versus 33.60 ± 2.55 min for the manual contouring of 28 OARs in head and neck cancer [24]. In a large multicenter cohort of HNC patients, auto-segmentation reduced the overall contouring time by 46% relative to manual delineation [25] while others reported up to a 90% reduction in the clinical workload with the use of AI for OAR delineation [26] or even higher [27]. In our study, a 56% time reduction was observed for the ten primary OARs, with the added benefit of additional OARs being contoured by the algorithm that conventionally are not included in the adopted protocol.

### 4.2. Performance Metrics

Across the group of primary OARs, a mean DSC value of 0.85 was achieved for each organ, meaning that 85% of the manually delineated volume was covered by the auto-segmentation across all organs. The oral cavity was the best recognized by the AI algorithm, at 91% coverage, while the thyroid gland had the lowest commonality between the manual and auto-segmentation, at 80%. The average 95% HD for all primary OARs was 4.46 mm, with the highest value recorded for the mandible (6.32 mm) and the lowest for the submandibular glands (right 3.17 mm, left 3.87 mm). The fact that the mandible achieved a relatively high 95% HD value and a 0.89 value for DSC (89% overlap between the manual and auto-segmentation) shows that high volume overlap and substantial boundary discrepancies can coexist. This means that most of the two segmented volumes overlap (high DSC) except for a few points that are grossly misjudged and determine the high value for the 95% HD parameter. High 95% HD values may also occur owing to dental hardware, which can reduce the interpretability of the CT images [28]. Moreover, organs with more regular shapes tend to be segmented by the AI within a lower 95% HD value, even if the DSC value is not among the highest, which demonstrates a systematic over- or under-segmentation compared to the ground truth. Organs that exhibit sharp bends in their anatomy tend to have higher 95% HD values, even while achieving a high DSC value, because of just a few points that do not fit the exact angling of the anatomy. While useful, judging a segmentation based purely on the 95% HD parameter is not advisable, as in some cases even a very small value might mean an overlap with a neighboring vital organ, while in other cases a large value might mean an intersection with a less important area. It should be noted that even the ground truth is not objectively “grounded”, especially the surface points of a segmentation may vary due to inter-observer differences, affecting the values of the DSC and 95% HD parameters.

Many of the above limitations, such as improving the value of DSC and reducing the value of 95% HD, are likely to be resolved by employing more performant AI algorithms such as transformer or foundation models (Segment Anything, META AI) [29,30].

### 4.3. Dosimetry of OARs

Unlike most previously published studies that focus predominantly on geometric performance metrics, the present work further investigates the clinical impact of segmentation differences by integrating a dosimetric evaluation of the OARs. This allows for a more meaningful clinical interpretation of segmentation performance, beyond pure geometric agreement.

Emerging evidence demonstrates that auto-segmentation often has a minimal impact on key dosimetric outcomes when used for routine planning, although correlations between geometric and dosimetric differences are not consistently strong across all organs and disease sites [31]. In our study, the dose differences between manual and auto-segmentation were found in the brainstem (4.5 Gy) and esophagus (4.7%). These discrepancies might be due to differences in the anatomical evaluation between the clinician and AI. For instance, the superior section of the brainstem shows a larger organ contour when delineated by AI, while the inferior part merges with the manually delineated spinal cord, often leading to overlaps or partial volume effects that influence the clinician’s delineation. For the esophagus, the discrepancies arise due to a lack of separation, resulting in the unintended inclusion of adjacent structures, such as the descending aorta and thoracic vertebrae, in the OAR contour.

Dose differences between the manual and auto-segmentation-based planning across other organs were similar to those reported in the literature. For the parotid glands, the geometric performance of the auto-segmentation led to similar dosimetric outcome to that reported by Gan et al. (2021) (3.7 ± 2.7 Gy) on a cohort of 15 CT images originating from HNC patients [32] but it was higher than the values reported by van Dijk et al. (2020) (0.9 ± 1.3 Gy) using their in-house neural network trained on 589 HNC patients and validated on 104 patients [33]. A recent evaluation of a RayStation DL model for the auto-segmentation of OARs in head and neck cancer reported differences between the manual and auto-segmentation within a ±3 Gy interval for most OARs on a dataset of 124 patients [34]. Exceptions were the esophagus and the larynx, where the mean dose differences were −5.9 Gy for the esophagus and increased up to 8.4 Gy for the larynx. These differences were attributed to variations in contouring between RayStation and the local protocol for these particular organs. The authors concluded that patient orientation, the degree of neck flexion and the presence of surgical implants, and postoperative anatomical changes are factors affecting the contouring outcomes, which further leads to dosimetric differences between the manual and auto-segmentation [34].

### 4.4. Secondary (Under-Reported) OARs

In addition to the ten commonly contoured OARs, the AI algorithm delineated 29 additional organs, allowing for an extended dosimetric evaluation. Given that the dosimetric differences between the ten manually and auto-contoured organs were clinically acceptable, it was considered safe to assume reliability concerning the secondary OARs. As shown in Table S2, for the 50 patients, 1500 dosimetric values were collected for these 29 organs only. It was shown that 128 dosimetric parameters exceeded the recommended dose constraints (8.5%) whereas 237 values (15.8%) were situated in the top 10% of the dose constraints (i.e., exceeded 90% of the highest recommended value), with these high values being spread over 15 organs. The organ with the highest number of patients (76%) over 90% of the constraint was the constrictor muscle, with 56% of patients exceeding the dose constraint for this organ. Four organs received more than 90% of the considered limit in over 50% of patients (glottis, constrictor muscle, cervical vertebrae, brachial plexus). In this cohort, the highest number of organs receiving over 90% of the constraint for the same patient was eight (two patients), while the highest number of organs over the dose constraint for the same patient was six (one patient). For only one patient were the doses for all secondary OARs found to be below 90% of the constraint.

Since no dose constraints have been established for the sublingual glands, both the mean (39.30 ± 16.79 Gy) and maximum doses (52.74 ± 16.44 Gy) were reported given the importance of these structures as organs at risk. These values exceed the dose constraints for the other salivary glands (parotid glands Dmean < 26 Gy and submandibular glands Dmean < 35 Gy). This raises a concern regarding the need to establish and validate a dose constraint for this OAR, which, along with the other two salivary glands, is responsible for radiation-induced xerostomia [35].

It is important to note that the observed dose constraint violations included both isolated cases and repeated occurrences across the cohort; however, no systematic distinction was made between sporadic outliers and frequent threshold exceedances. From a clinical perspective, this differentiation is relevant, as isolated violations may have limited clinical relevance, whereas recurrent deviations could indicate systematic planning or delineation challenges. Given the retrospective nature of this study, all patients had already completed radiotherapy prior to the use of the auto-segmentation software (Mediq RT, Synaptiq). Therefore, these dosimetric findings were not part of the original clinical decision-making process and did not influence treatment planning or adaptive strategies. Nevertheless, their presence highlights the importance of carefully evaluating under-reported or highly sensitive OARs, as they may contribute to a more comprehensive understanding of plan quality and potential areas for improvement in contouring and dose optimization.

In this context, the auto-segmentation of the primary OARs required, in most cases, minimal intervention from the operator, as supported by the comparison with manual delineations. In contrast, the segmentation of the secondary OARs provides additional dosimetric information; however, its clinical interpretation should be considered exploratory, as no dedicated manual reference contours were available for validation in this group. Table S2 illustrates that the doses received by the secondary OARs should be examined within the treatment plan to ensure compliance with the dose constraints.

### 4.5. Toxicity Assessment

The inclusion of the secondary OARs enables a more comprehensive assessment of radiation exposure, which may provide valuable insights into potential toxicity risks and their impact on patients’ quality of life.

In the present study, weak-to-moderate correlations were observed between the radiation dose to the secondary OARs and the treatment-related toxicities. Xerostomia demonstrated a moderate correlation with the sublingual gland Dmean (r = 0.449) and a weak correlation with the Dmax (r = 0.347). However, current dose–response evidence regarding radiation-induced xerostomia is primarily focused on the parotid and submandibular glands, while data regarding the sublingual glands remains limited. Therefore, these findings should be interpreted as hypothesis-generating and may indicate the need for further investigation of under-reported salivary structures in head and neck radiotherapy.

Similarly, weak correlations were observed between dysphagia and the dose to the constrictor muscles (r = 0.252) and the supraglottic larynx (r = 0.241), while odynophagia showed slightly higher correlations with the supraglottic larynx dose (r = 0.388) and the constrictor muscle dose (r = 0.280). These findings indicate weak associations between the dose to swallowing-related structures and the reported toxicities, which may be partly explained by the limited sample size and the multifactorial nature of radiation-induced symptoms. Importantly, such weak correlations should not be interpreted as clinically irrelevant, as previous prospective studies have demonstrated that the dose to midline swallowing structures, including the pharyngeal constrictors and laryngeal components, is significantly associated with the severity of dysphagia during and after intensity-modulated radiotherapy. In particular, Kannan et al. reported that higher radiation doses to swallowing structures correlate with impaired functional recovery and that limiting the dose to approximately 45 Gy was associated with improved swallowing outcomes and reduced long-term dysphagia, thereby underscoring the clinical relevance of maintaining dose constraints for dysphagia-related structures [36].

Despite these correlations, the modest coefficients likely reflect the multifactorial nature of radiation-induced toxicities, which are influenced by tumor location, baseline swallowing status, and patient-specific anatomy. Nevertheless, our results support the potential clinical relevance of dose exceedances in these structures, highlighting the importance of careful treatment planning to minimize the dose to secondary OARs. Future prospective studies integrating dosimetric, clinical, and patient-reported outcomes are warranted to further clarify these relationships.

### 4.6. Limitations of the Study

In the current study, the ground truth was provided by the clinician and an experienced medical physicist assigned to each patient; however, no quantitative assessment of inter-observer variability was performed. The auto-segmentation algorithm was trained on a relatively small dataset of 7000 CT images. At times, the algorithm exhibited difficulties in contouring certain secondary OARs, such as the glottis and the brachial plexus, which could be due to the low CT contrast, tumor location and inherent challenges in delineating complex anatomical structures [37,38]. The number of patients included in this study was small-to-moderate (50 patients); however, the study remains relevant, as the cohort remained homogeneous due to the strict inclusion and exclusion criteria. Furthermore, the obtained results may support and justify the development of future clinical studies involving larger patient cohorts. The different tumor locations within the HNC patient cohort are reflected in the variability of the performance metrics used for the comparison between the auto- and manual segmentation, as well as in the dose received by the adjacent OARs, particularly when comparing nasopharyngeal with laryngeal tumors, which may contribute to variability in the dosimetric outcomes and compliance with the dose constraints. Nevertheless, tumor location did not appear to influence segmentation accuracy. For some patients, certain OARs were partially included in the treated volume.

### 4.7. Challenges and Future Perspectives

Recent systematic evaluations comparing multiple commercial auto-contouring solutions have highlighted that while the overall segmentation quality is acceptable for many OARs, performance varies by organ and clinical context. Complex structures such as the optic chiasm or regions affected by imaging artifacts still frequently require manual refinement despite the use of advanced algorithms [39], as variability in training datasets, contouring protocols, and ground truth definitions continues to limit generalizability.

Another emerging area of research in this field is the integration of multimodal imaging approaches. While most algorithms are developed using CT images, MRI and PET provide complementary soft tissue contrast that can enhance segmentation for certain OARs [40].

Better quality assurance frameworks, interpretability of AI outputs, and prospective clinical trials are needed to accelerate the adoption and regulatory acceptance of auto-segmentation. Evaluation beyond geometric metrics, including dosimetric and clinical endpoints, will be crucial for wider clinical integration. Despite technological advances, accurate and reliable OAR contouring in head and neck radiotherapy continues to require a hybrid approach that combines automated tools with clinician oversight to ensure patient safety and treatment quality [12,13].

## 5. Conclusions

Auto-segmentation methods in radiotherapy planning substantially improve time efficiency compared with conventional manual segmentation, even accounting for the time spent adjusting the auto-segmented results. Once validated for primary OAR segmentation quality, auto-segmentation can be considered for secondary OARs. This enables a dosimetric evaluation of all organs at risk, thereby minimizing possible side effects and improving the quality of life of head and neck cancer patients.

## Figures and Tables

**Table 1:** Dose constraints for primary and secondary organs at risk [16,17].

OARs	Dose Constraint
Primary (conventional) OARs	
Brainstem	Dmax < 54 Gy
Spinal cord	Dmax < 45 Gy
Esophagus	V35 < 50%
Thyroid gland	V45 < 50%
Submandibular glands	Dmean < 35 Gy
Parotid glands	Dmean < 26 Gy
Oral cavity	Dmean < 30 Gy
Mandible	Dmax < 72 Gy
Secondary (under-reported) OARs	
Brain	Dmax < 60 Gy
Temporal lobes	Dmax < 58 Gy
Optic chiasm	Dmax < 54 Gy
Pituitary gland	Dmax < 45 Gy
Internal ears	Dmax < 50 Gy
Cochleas	Dmean < 45 Gy
Eyes	Dmean < 35 Gy
Lenses	Dmax < 7 Gy
Optic nerves	Dmax < 54 Gy
Lacrimal glands	Dmean < 25 Gy
Cervical vertebrae	ALARA
Sublingual glands	ALARA
Larynx	V50 < 25%
Larynx-supraglottis	Dmax < 66 Gy
Glottis	Dmean < 45 Gy
Trachea	V40 < 30%
Clavicles	ALARA
TM	Dmax > 70 Gy not to exceed 0.1 cm^3^
Constrictor muscle	Dmean < 50 Gy
Brachial plexus	Dmax < 66–70 Gy

Abbreviations: OARs = organs at risk; TM = Temporomandibular joints; ALARA = as low as reasonably achievable; Dmean = mean dose; Dmax = maximum dose; Vx = % of volume receiving more than x Gy.

**Table 2:** Mean delineation times for manual, auto- and adjusted auto-segmentation methods.

Segmentation and Adjustment Time	Manual Segmentation (min.sec) *	Auto-Segmentation (min.sec) **	Adjusted Auto-Segmentation (min.sec) ***	Auto-Segmentation + Adjusted Auto-Segmentation (min.sec)
Mean ± SD	21.20 ± 2.25	0.33 ± 0.11	8.52 ± 1.37	9.25 ± 1.42

SD = standard deviation; * Time taken for manually contoured organs at risk (primary OARs); ** Time taken by auto-segmentation for all organs at risk (primary OARs + secondary OARs); *** Time taken to adjust primary OARs contoured by auto-segmentation against manual segmentation.

**Table 3:** Mean values and standard deviations of the performance metrics—Hausdorff distance, Dice Similarity Coefficient, sensitivity and precision—for the ten primary organs at risk.

Primary OARs	Max HD (mm ± SD)	95% HD (mm ± SD)	DSC (±SD)	Sensitivity (±SD)	Precision (±SD)
Spinal cord	8.33 ± 7.87	3.23 ± 3.15	0.83 ± 0.03	0.85 ± 0.08	0.83 ± 0.08
Oral cavity	9.33 ± 4.38	4.91 ± 3.15	0.91 ± 0.03	0.88 ± 0.08	0.87 ± 0.05
Right submandibular gland	6.58 ± 3.60	3.17 ± 1.50	0.86 ± 0.04	0.83 ± 0.08	0.90 ± 0.05
Left submandibular gland	7.28 ± 5.54	3.87 ± 2.91	0.84 ± 0.09	0.81 ± 0.11	0.87 ± 0.09
Right parotid	10.07 ± 5.87	4.57 ± 2.66	0.87 ± 0.07	0.85 ± 0.09	0.91 ± 0.06
Left parotid	9.27 ± 4.24	4.44 ± 2.39	0.86 ± 0.06	0.86 ± 0.09	0.87 ± 0.08
Brainstem	9.76 ± 3.21	5.80 ± 2.34	0.81 ± 0.06	0.80 ± 0.09	0.84 ± 0.10
Mandible	24.09 ± 18.83	6.32 ± 8.40	0.89 ± 0.12	0.87 ± 0.14	0.93 ± 0.05
Esophagus	9.13 ± 5.34	4.28 ± 2.66	0.82 ± 0.04	0.77 ± 0.06	0.88 ± 0.05
Thyroid gland	10.24 ± 4.76	4.05 ± 2.61	0.80 ± 0.07	0.81 ± 0.08	0.81 ± 0.11

Abbreviations: Max HD = maximum Hausdorff distance; 95% HD = 95% of surface points are within the given value; SD = standard deviation; DSC = Dice Similarity Coefficient.

**Table 4:** Pearson coefficient calculated between dose differences and performance metrics (a strong correlation was highlighted in bold orange, while a moderate correlation was highlighted in bold blue).

Pearson Coefficient	Max HD	95% HD	DSC	Sensitivity	Precision
Spinal cord	0.017	−0.110	0.062	0.317	−0.295
Oral cavity	**−0.747**	**−0.795**	**0.465**	0.299	0.041
Right submandibular gland	0.006	0.029	−0.065	−0.152	0.107
Left submandibular gland	0.117	0.140	−0.043	0.047	−0.153
Right parotid	0.008	−0.126	**0.511**	**0.613**	−0.254
Left parotid	−0.087	−0.156	0.314	**0.476**	−0.295
Brainstem	0.242	0.269	−0.385	0.143	**−0.611**
Mandible	0.124	0.094	−0.086	−0.102	0.024
Esophagus	0.023	0.110	−0.262	−0.322	−0.080
Thyroid gland	−0.235	−0.089	0.091	0.178	−0.005

Abbreviations: Max HD = maximum Hausdorff distance; 95% = measures the 95% distance between the surface points of one object and their corresponding points in the other object; DSC = Dice Similarity Coefficient.

**Table 5:** Mean dose and the highest dose for secondary organs at risk (doses exceeding the constraints are highlighted in bold blue).

Organ at Risk	Mean Dose ± SD	Highest Dose Out of All 50 Patients
Brain	34.51 ± 12.59 Gy	58.17
Right internal ear	27.96 ± 20.82 Gy	**68.79**
Left internal ear	27.25 ± 20.63 Gy	**59.04**
Right eye	0.82 ± 1.26 Gy	9.28
Left eye	0.75 ± 0.80 Gy	5.90
Right lacrimal gland	0.59 ± 0.51 Gy	3.76
Left lacrimal gland	0.57 ± 0.46 Gy	3.35
Sublingual glands *	39.30 ± 16.79 Gy	69.58
	52.74 ± 16.44 Gy	72.75
Right lens	0.89 ± 0.90 Gy	6.41
Left lens	0.84 ± 0.70 Gy	4.90
Right temporal lobe	4.34 ± 7.29 Gy	47.51
Left temporal lobe	5.15 ± 8.70 Gy	52.52
Right optic nerve	2.31 ± 8.07 Gy	**58.08**
Left optic nerve	2.26 ± 7.89 Gy	**56.79**
Optic chiasm	2.10 ± 6.83 Gy	49.29
Pituitary	2.69 ± 6.80 Gy	**49.20**
Larynx **	**27.15 ** **± 39.03%**	**100**
Larynx-supraglottis	64.70 ± 8.38 Gy	**77.15**
Glottis	**51.53 ** **± 15.02 Gy**	**68.73**
Right clavicle	49.20 ± 12.18 Gy	62.37
Left clavicle	50.62 ± 12.71 Gy	67.25
Right TM joint	13.00 ± 13.05 Gy	56.18
Left TM joint	12.43 ± 12.32 Gy	46.48
Constrictor muscle	**49.90 ** **± 6.59 Gy**	**61.71**
Trachea	18.23 ± 13.43%	**53.50**
Cervical vertebrae	63.95 ± 4.33 Gy	70.78
Right cochlea	4.61 ± 5.07 Gy	23.88
Left cochlea	4.52 ± 5.48 Gy	23.82
Brachial plexus	60.77 ± 5.73 Gy	**70.43**

Abbreviations: SD = standard deviation; TM = temporomandibular; * The first row corresponds to the mean dose, and the second row corresponds to the maximum dose. ** To be noted that the larynx was a target volume in 28 instances and an OAR in 22 cases.

## Data Availability

The original contributions presented in this study are included in the article/Supplementary Material. Further inquiries can be directed to the corresponding author.

## Supplementary Materials

The following supporting information can be downloaded at: https://www.mdpi.com/article/10.3390/diagnostics16111748/s1, Table S1: Patient characteristics; Table S2: Evaluated dosimetry of secondary organs at risk for all patients; Table S3: Correlations between treatment-related toxicities and dosimetry of related secondary OARs; Figure S1: Box plots of maximum HD and 95% HD for the 10 primary organs at risk; Figure S2: Box plots of performance metrics: Dice Similarity Coefficient, sensitivity and precision) for the ten primary organs at risk.
