# Supplementary material for: AI-Based Dose Compliance of Secondary Organs at Risk in Head and Neck Cancer Radiotherapy
Source: Diagnostics (Basel). 2026 Jun 5;16(11):1748. doi: 10.3390/diagnostics16111748 (PMC13256923; doi:10.3390/diagnostics16111748)
Supplement: Supplementary file 1 [file diagnostics-16-01748-s001.zip › Table S1.pdf]

Table S1. Patient characteristics

Patient	Age	Stage	Tumor localization	Dose prescription
1	48	$cT_2N_{2c}M_0$	pyriform sinus scc.	50/25 + boost 60/30 and 70/35
2	51	$cT_4N_{2c}M_0$	hypopharyngeal scc.	56/63/70 in 35 fr
3	66	$pT_{4a}N_0M_0$	interstitial adc. of left nasal fossa with extension into the sphenoid sinus	50/25 + boost 60/30 and 70/35
4	67	$cT_2N_{2c}M_0$	pyriform sinus scc.	50/25 + boost 60/30 and 70/35
5	57	$T_4N_{2c}M_0$	laryngopharyngeal scc.	50/25 + boost 60/30 and 70/35
6	57	N/A	pyriform sinus scc.	50/25 + boost 60/30 and 70/35
7	79	$cT_3N_0M_0$	larynx glottis scc.	56/63/70 in 35 fr
8	72	$T_3N_0M_0$	left glosso-epiglottic keratinized scc.	56/63/70 in 35 fr
9	52	$cT_{4b}N_3M_0$	undifferentiated pyriform sinus c.	56/63/70 in 35 fr
10	53	$cT_4N_0M_0$	larynx scc.	50/25 + boost 60/30 and 70/35
11	70	$cT_4N_2M_0$	hemitongue scc.	50/25 + boost 60/30 and 70/35
12	74	N/A	base of tongue scc.	50/25 + boost 60/30 and 70/35
13	76	$cT_2N_{2c}M_0$	left tonsillar fossa scc.	50/25 + boost 60/30 and 70/35
14	49	$cT_2N_{2c}M_0$	larynx supraglottis scc.	50/25 + boost 60/30 and 70/35
15	69	$cT_3N_{2b}M_0$	hemitongue scc.	50/25 + boost 60/30 and 70/35
16	72	N/A	scc. of inferior lip	50/25 + boost 60/30 and 70/35
17	57	$cT_3N_{2b}M_0$	base of tongue scc.	50/25 + boost 60/30 and 70/35
18	64	$cT_4N_{2c}M_0$	left pyriform sinus scc.	50/25 + boost 60/30 and 70/35
19	80	$cT_4N_0M_0$	larynx glottis scc.	50/25 + boost 60/30 and 70/35
20	77	$cT_3N_0M_0$	larynx scc.	50/25 + boost 60/30 and 70/35
21	49	N/A	keratinized scc. of the right lingual vallecula	56/63/70 in 35 fr
22	71	$cT_3N_{2c}M_0$	scc. of the retromolar trigone	56/63/70 in 35 fr
23	52	N/A	keratinized scc. of the retromolar trigone	56/63/70 in 35 fr
24	71	$cT_2N_0M_0$	larynx glottis scc.	56/63/70 in 35 fr
25	57	$cT_4N_2M_0$	left base of tongue scc.	50/25 + boost 60/30 and 70/35
26	57	$cT_4N_2M_0$	oral cavity palate scc.	56/63/70 in 35 fr
27	60	$cT_4N_{2b}M_0$	laryngopharyngeal scc.	50/25 + boost 60/30 and 70/35
28	59	$cT_4N_{2c}M_0$	base of tongue scc.	56/63/70 in 35 fr
29	50	N/A	pyriform sinus scc.	56/63/70 in 35 fr
30	51	$pT_2N_{2b}M_0$	hemitongue scc.	50/25 + boost 60/30 and 70/35
31	75	$cT_2N_0M_0$	pyriform sinus scc.	56/63/70 in 35 fr
32	56	$cT_3N_{2c}M_0$	larynx supraglottis scc.	56/63/70 in 35 fr
33	73	$cT_3N_0M_0$	larynx glottis scc.	56/63/70 in 35 fr
34	75	$cT_3N_0M_0$	pyriform sinus scc.	56/63/70 in 35 fr
35	51	$cT_2N_{2c}M_0$	oral cavity scc.	56/63/70 in 35 fr
36	68	$cT_4N_{2c}M_0$	oropharyngeal scc.	56/63/70 in 35 fr

37	71	$cT_3N_{2c}M_0$	base of tongue scc.	56/63/70 in 35 fr
38	62	$cT_3N_{2c}M_0$	pyriform sinus scc.	56/63/70 in 35 fr
39	64	$cT_2N_{2b}M_0$	oral cavity palate scc.	56/63/70 in 35 fr
40	61	$pT_3N_0M_0$	larynx glottis scc.	56/63/70 in 35 fr
41	63	$cT_3N_3M_0$	pyriform sinus scc.	56/63/70 in 35 fr
42	65	$cT_4N_0M_0$	hemitongue scc.	56/63/70 in 35 fr
43	76	$cT_3N_{2b}M_0$	larynx supraglottis scc.	56/63/70 in 35 fr
44	59	$cT_4N_2M_0$	larynx supraglottis scc.	56/63/70 in 35 fr
45	70	N/A	larynx glottis scc.	50/25 + boost 60/30 and 70/35
46	61	$cT_2N_{3a}M_0$	epiglottis scc.	50/25 + boost 60/30 and 70/35
47	58	$cT_3N_{2b}M_0$	pyriform sinus scc.	50/25 + boost 60/30 and 70/35
48	89	N/A	right tonsil scc.	56/63/70 in 35 fr
49	81	$cT_2N_0M_0$	left hypopharyngeal scc.	50/25 + boost 60/30 and 70/35
50	67	$cT_2N_0M_0$	supraglottis scc.	50/25 + boost 60/30 and 70/35
**Mean**	**64.24**			

Abbreviations: scc. = squamous cell carcinoma; c. = carcinoma; adc. = adenocarcinoma; fr = fractions, N/A = not available.
