# Supplementary material for: AI-Based Dose Compliance of Secondary Organs at Risk in Head and Neck Cancer Radiotherapy
Source: Diagnostics (Basel). 2026 Jun 5;16(11):1748. doi: 10.3390/diagnostics16111748 (PMC13256923; doi:10.3390/diagnostics16111748)
Supplement: Supplementary file 1 [file diagnostics-16-01748-s001.zip › Table S3.pdf]

Table S3. Correlations between treatment-related toxicities and dosimetry of related secondary OARs

Toxicity	Secondary OAR	r coefficient*
Xerostomia	Sublingual glands Dmean	0.449
	Sublingual glands Dmax	0.347
Dysphagia	Constrictor muscle	0.252
	Larynx-supraglottis	0.241
Odynophagia	Constrictor muscle	0.280
	Larynx-supraglottis	0.388

Abbreviation: Dmean = mean dose; Dmax = maximum dose; OAR = organ at risk.
*Pearson correlation coefficient
