# Supplementary figures and images for: AI-Based Dose Compliance of Secondary Organs at Risk in Head and Neck Cancer Radiotherapy
Source: Diagnostics (Basel). 2026 Jun 5;16(11):1748. doi: 10.3390/diagnostics16111748 (PMC13256923; doi:10.3390/diagnostics16111748)

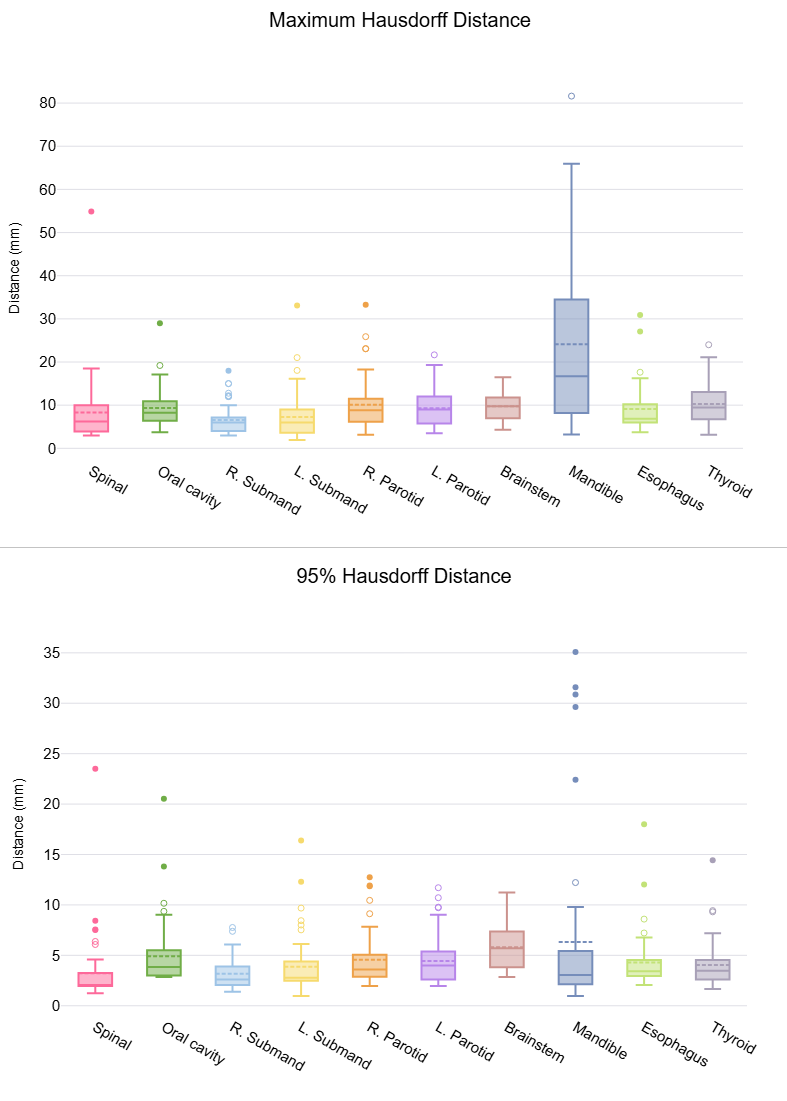

Supplement: Supplementary file 1 [file diagnostics-16-01748-s001.zip › Figure S1.png]

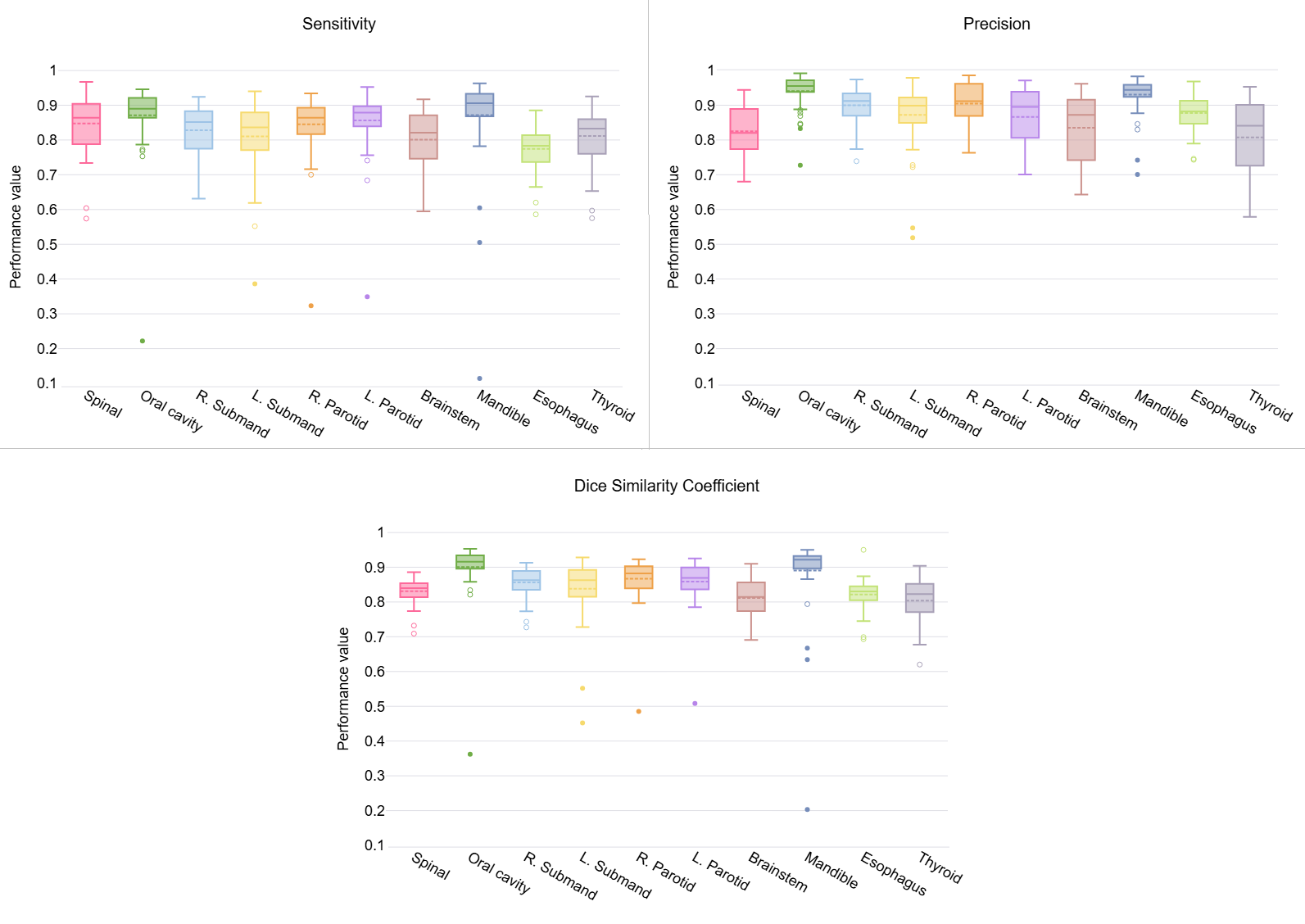

Supplement: Supplementary file 1 [file diagnostics-16-01748-s001.zip › Figure S2.png]
